# Antifungal activity of liriodenine on clinical strains of *Cryptococcus neoformans* and *Cryptococcus gattii* species complexes

**DOI:** 10.1590/1678-9199-JVATITD-2022-0006

**Published:** 2022-09-05

**Authors:** Adriele Dandara Levorato-Vinche, Marcia de Souza Carvalho Melhem, Lucas Xavier Bonfietti, Iván de-la-Cruz-Chacón, Carmen Sílvia Fernandes Boaro, Alexandre Todorovic Fabro, Gisela Ferreira, Julhiany de Fátima da Silva, Daniela Carvalho dos Santos, Beatriz Aparecida Soares Pereira, Camila Marçon, Lariza Maza, Lídia Raquel de Carvalho, Rinaldo Poncio Mendes

**Affiliations:** 1 Department of Infectology, Dermatology, Diagnostic Imaging and Radiotherapy, Botucatu Medical School (FMB), São Paulo State University (UNESP), Botucatu, SP, Brazil.; 2 Mycology Unit, Adolfo Lutz Institute, Public Health Reference Laboratory, Secretariat of Health of the State of São Paulo, São Paulo, SP, Brazil.; 3 Medical School, Federal University of Mato Grosso do Sul, Campo Grande, MS, Brazil.; 4 Instituto de Ciencias Biológicas, Universidad de Ciencias y Artes de Chiapas, Tuxtla Gutierrez, Chiapas, Mexico.; 5 Department of Biostatistics, Plant Biology, Parasitology and Zoology, Botucatu Biosciences Institute, São Paulo State University (UNESP), Botucatu, SP, Brazil.; 6 Department of Pathology and Legal Medicine, Ribeirão Preto Medical School, University of São Paulo (USP), Ribeirão Preto, SP, Brazil.; 7 Department of Structural and Functional Biology, Botucatu Biosciences Institute, São Paulo State University (UNESP), Botucatu, SP, Brazil.

**Keywords:** Liriodenine, Yeast, Fungi, Fungal cell ultrastructure, *Cryptococcus* spp., Time-kill methodology

## Abstract

**Background:**

Cryptoccocal meningitis continues to present high incidence among AIDS patients. The treatment of choice is the synergistic combination of flucytosine (5-FC) with amphotericin B deoxycholate (AmBd) or its lipid formulations. However, 5-FC is unavailable in many countries and AmB demands hospitalization. The combination of AmB with the fungistatic fluconazole (FLC) or the use of high FLC daily doses alone became the choice. Nonetheless, sterilization of cerebrospinal fluid is delayed with FLC monotherapy, mainly with high fungal burden. These findings suggest the search for new antifungal compounds, such as liriodenine.

**Methods:**

Liriodenine antifungal activity was evaluated by three procedures: determining the minimum inhibitory concentration (MIC) on 30 strains of the *Cryptococcus neoformans* (*C. neoformans*) complex and 30 of the *Cryptococcus gattii* (*C. gattii*) complex, using EUCAST methodology and amphotericin B deoxycholate as control; performing the time-kill methodology in two strains of the *C. neoformans* complex and one of the *C. gattii* complex; and injury to cryptococcal cells, evaluated by transmission electron microscopy (TEM). Liriodenine absorption and safety at 0.75 and 1.50 mg.kg^-1^ doses were evaluated in BALB/c mice.

**Results:**

Liriodenine MICs ranged from 3.9 to 62.5 μg.mL^-1^ for both species complexes, with no differences between them. Time-kill methodology confirmed its concentration-dependent fungicidal effect, killing all the strains below the limit of detection (33 CFU.mL^-1^) at the highest liriodenine concentration (32-fold MIC), with predominant activity during the first 48 hours. Liriodenine induced severe *Cryptococcus* alterations - cytoplasm with intense rarefaction and/or degradation, injury of organelles, and presence of vacuoles. Liriodenine was better absorbed at lower doses, with no histopathological alterations on the digestive tract.

**Conclusion:**

The fungicidal activity confirmed by time-kill methodology, the intense *Cryptococcus* injury observed by TEM, the absorption after gavage administration, and the safety at the tested doses indicate that the liriodenine molecule is a promising drug lead for development of anticryptococcal agents.

## Background

The increased incidence of fungal infections, mainly in immunosuppressed patients, and the emergence of resistant relevant isolates in many countries worldwide for all available compounds, except amphotericin B (AmB), demonstrate the importance of looking for new antifungal compounds, mainly those recommended for treatment of systemic diseases [1, 2]. 

Cryptococcosis is a systemic mycosis caused by encapsulated and non-fermentative yeasts of the *Cryptococcus neoformans* (*C. neoformans*) and *Cryptococcus gattii* (*C. gattii*) species complexes [3]. The lungs are the primary portal of entry and focus of this infection, but life-threatening meningitis is its main clinical manifestation, including in AIDS patients [4, 5].

Treatment of cryptococcal meningitis is carried out with the synergistic combination of flucytosine with AmBd or its lipid formulations. Nonetheless, flucytosine is unavailable in many countries, and AmB demands hospitalization, leading to the use of the combination AmB and fluconazole (FLC) for the initial treatment in several countries such as Brazil, or the use of higher doses of FLC as it is in many African countries [6]. Since FLC is fungistatic and causes a slow clearance of the fungus, other regimens and/or combinations with immunomodulators have been evaluated [6-8].

A number of plants produce molecules with antimicrobial activity, including several species of the Annonaceae family, which produce benzylisoquinoline alkaloids [9]. One of the most common ones is liriodenine, an alkaloid aporphine type found in around 90 genera and 300 Annonaceae species [10, 11] with activity on fungi, bacteria, and protozoa [12-17]. 

Levorato-Vinche et al. [18] evaluated the *in vitro* antifungal activity of liriodenine on agents of systemic mycoses, and it showed a minimum inhibitory concentration (MIC) of 62.5 µg.mL^-1^ on most of the isolates. Moreover, its activity was fungicidal on susceptible isolates. 

The present study aimed at evaluating liriodenine antifungal activity on isolates of both *C. neoformans* and *C. gattii* species complexes using the time-kill curve and the analysis of cell alterations by electron microscopy. 

## Methods

### Antifungal compounds

AmBd (Sigma Chemical Company, St. Louis, MO, USA) and liriodenine were used for MIC testing. Moreover, a time-kill evaluation of liriodenine was performed.

### Source and extraction of liriodenine

Liriodenine was obtained from *Annona mucosa* Jacq. planted at the geographical coordinates 22º59’27’’S and 48º28’22’’W in Rio Sul locality, Botucatu (São Paulo state, Brazil). The taxonomic sample was under reference voucher number 33185 of the BOTU Herbarium - São Paulo State University (UNESP), Botucatu (São Paulo state, Brazil). This species is neither threatened nor protected. Liriodenine is an oxoaporphine alkaloid, yellow needle, fluorescent, plain, with an oxo group in position 7, isolated from the root barks.

Liriodenine was extracted from root bark (1,000 g) in the Laboratory of Botany of Botucatu Biosciences Institute, São Paulo State University (UNESP), Brazil, according to a previously reported methodology [19].

### Stock solutions

Stock solutions of each agent were prepared using dimethyl sulfoxide (DMSO). Stock solutions of 1000 μg.mL^-1^ were separated into aliquots and stored at -70ºC until they were used. RPMI 1640 liquid medium (Sigma) buffered to a pH 7.0 with 0.165 M morpholinepropanesulfonic acid (Sigma) was used to obtain final tested agent concentrations. To demonstrate that DMSO did not affect the growth of the studied strains, fungal colonies were grown in the presence of final (1% vol/vol) DMSO concentration and compared to growth in DMSO-naïve conditions. 

### Microorganisms

Sixty strains, previously identified by molecular typing [20], were evaluated in this study. Thirty of them belonged to the *C. neoformans* complex and the other 30 to the *C. gattii* complex. Regarding AFST-EUCAST recommendations, two quality control strains - *Candida krusei* (ATCC 6258) and *Candida parapsilosis* (ATCC 22019) - were also evaluated along with the tests. More than 99% of the QC results were within the acceptable limits. These isolates were kept frozen in 15% glycerol solutions at -20 °C at the Laboratory of Tropical Diseases, Section of Medical Mycology, Experimental Research Unit (Unipex), Botucatu Medical School, UNESP, until they were ready for use in the study. Before testing, each strain was plated on CHROMagar *Candida®* (Becton Dickinson, Franklin Lakes, NJ, USA) to ensure purity and viability.

### Cryptococcal susceptibility testing

 The *in vitro* susceptibility to liriodenine and to AmBd was evaluated by the broth microdilution method, according to the European Committee on Susceptibility Testing (EUCAST) [21]. The antifungal epidemiologic cutoff values (ECOFFs) and clinical breakpoints were proposed according to the EUCAST E.Def 7.3 document (European Committee on Antimicrobial Susceptibility Testing), and by using colorimetric indicators [22].

Serial dilutions of the stock solution using RPMI-1640 as diluent were performed, and 10 concentrations of liriodenine ranging from 0.97 to 500 μg.mL^-1^ were obtained. Volumes of 100 μL of each concentration were distributed in microplates with 96 wells. AmBd was included as a positive control in 10 concentrations from 0.03 to 16 μg.mL^-1^. The inocula of the fungal cells were adjusted to a final concentration of 1-5 × 10^5^ cells/mL, and 100 μL were added to each well containing either liriodenine or AmBd, and to the control wells without antifungal compounds. The plates were incubated at 35 (C ± 2 ºC for 48 h, and then the reading was performed with a microdilution plate reader under 450 nm wavelength. The antifungal concentration that elicited an relative absorbance ≤ 50% or < 90% of that observed in the negative control well (without antifungal compound) was considered the MIC of liriodenine and AmBd, respectively. All the tests were carried out in triplicate [21].

### Time-kill studies with liriodenine

The time-kill studies were conducted with two strains (strain 32 and strain 57) of the *C. neoformans* complex and one (strain 17) of the *C. gattii* complex, according to the procedures of Klepser et al. [23] and Silva et al. [24]. Initially, the isolates were subcultured on potato dextrose agar plates. Individual colonies (≥ 1mm) from 48-h culture were suspended in 10-mL buffered RPMI 1640 with 2 % glucose and L-glutamine medium. Isolates were grown overnight with shaking at 35 °C. The initial inoculum was adjusted to 0.5 McFarland turbidity standard (10^6^ CFU.mL^-1^). One milliliter of the adjusted fungal suspension was then added to either a 9-mL MOPS-buffered RPMI medium alone (control) or a solution of culture medium containing liriodenine. Liriodenine was tested in eight concentrations calculated as multiples of the MIC values (0.5×, 1×, 2×, 3×, 4×, 8×, 16× and 32× MIC). The test tubes were incubated at 35 (C ± 2 °C under agitation. Fungal growth was monitored over a time-course of 72 h (0, 2, 4, 6, 8, 12, 24, 48, 72 h). For every sampled time point, 0.5 mL of the tube content was removed, serially diluted 1:10 in sterile deionized water, and viable counts were determined in 30 μL plated on Sabouraud dextrose agar [25-27].

### Analysis

 The data were analyzed according to Klepser et al [23]. Colony counting data, in log_10_ CFU per milliliter, from time-kill studies in triplicate, were averaged and plotted as function of time for each isolate. The rate and extent of liriodenine antifungal activity were assessed. Fungicidal activity was defined as a ≥ 99.9% reduction in the number of CFU per milliliter from the starting inoculum count, and fungistatic activity occurred when this decrease was < 99.9%. 

### Transmission electron microscopy

Transmission electron microscopy (TEM) was performed at the Center of Electron Microscopy of São Paulo State University (UNESP), Botucatu Biosciences Institute (São Paulo state, Brazil), regarding its previously reported specifications [18].

Two strains, numbered 21 (*C. gattii*) and 41 (*C. neoformans)*, were treated with the respective liriodenine MICs in a 24-well plate. The final volumes of liriodenine and inoculum were adjusted to 1 mL, and Karnovsky’s fixative was added after 48-h incubation at 37 °C. Following this period, the material was removed from the fixative and washed three times for 5 min each in 0.1 M phosphate buffer, pH 7.3. The material was immersed in 0.1 M osmium tetroxide, pH 7.3, for 2 h. Next, the material was washed three times for 10 min each in distilled water and immersed in 0.5% uranyl acetate for approximately 2 h. After dehydration in an increasing acetone series, a mixture of Araldite® resin + 100% acetone (1:1) was added, and the material was left to stand at room temperature for 12 h. Pure resin was added for approximately one hour at 37ºC, and the material was embedded. Ultrathin (90 nm) sections were cut from the blocks and counterstained with uranyl acetate in 50% alcohol for 20 min, followed by counterstaining with lead citrate for 10 min. The sections were observed with a Tecnai Spirit transmission electron microscope (FEI Company).

### Statistical analysis

The comparison between the MIC values of different species and the antifungal compounds was carried out using the Mann-Whitney test. The correlation of MIC between antifungal compounds was performed by the Spearman rank correlation coefficient. Statistical tests were performed by using SAS (SAS Institute, Cary, NC, USA). Significance was set up at *p* ≤ 0.05. 

### *In vivo* evaluation of absorption and toxicity

*Animals*

Four isogenic albino male BALB/c mice, five to seven weeks of age and 25 g average weight, were obtained from the husbandry of the Experimental Laboratory of Infectious and Parasitic Diseases of Botucatu Medical School, São Paulo State University (UNESP), Botucatu (São Paulo state, Brazil). The animals were kept in boxes containing two animals on average, with a bed of pressure-treated wood shavings, in an environment ranging 23-25 °C, with lighting controlled by electromechanical time switch, that is, 12 h with the light on and 12 h in the dark. They received filtered water and commercial mice diet *ad libitum*.

*Liriodenine preparation and administration in uninfected mice*

Liriodenine was dissolved in 4% DMSO. For the preparation of the doses 0.75 and 1.50 mg.kg^-1^, 1.5 and 3.0 mg of liriodenine, respectively, 0.240 mL of 4% DMSO were added. Liriodenine was given by gavage in a single dose at 12:00a.m. Each animal received 0.120 mL of the liriodenine-containing solution. The doses used were defined based on the results of the *in vitro* sensitivity test [18]. Two uninfected mice received 0.75 mg.kg^-1^ and other two 1.50 mg.kg^-1^ of liriodenine in a single dose.

*Blood collection and euthanasia of the mice*

The animals were submitted to blood collection and euthanasia six and 12 h after liriodenine administration. Blood samples were collected from each animal by cardiac puncture to determine the serum levels of liriodenine. 

Mice were initially anesthetized and killed with 0.08 μL of a solution containing 24 μL ketamine, 32 μL xylosin, and 24 μL distilled water, which was administered intraperitoneally with the aid of a 1.0 mL disposable syringe. After sedation, the blood was collected by cardiac puncture using a 1.0 mL syringe and a 25 × 7-gauge needle. The blood was transferred to a sterile test tube without anticoagulants and, subsequently, centrifuged at 3.500 rpm in a Revan Cycle C I centrifuge.

*Serum dosage of liriodenine*

The serum samples from the mice were diluted in 15 mL deionized water to increase the volume of the working solution. In order to extract the fat present in the serum, the mixture was placed in a separatory funnel with 10 mL hexane, from which the aqueous phase was collected and then adjusted to 9.5 pH with saturated solution of Na_2_CO_3_. After that, it was placed in a separatory funnel with 10 mL chloroform, and the chloroform phase containing the liriodenine was then collected. For the identification and quantification of the liriodenine, the sample was kept to stand until the chloroform was completely evaporated, then dissolved in methanol and analyzed in High Performance Liquid Chromatography (HPLC), according to the specifications modified by de-La-Cruz-Chacón et al. [16, 17].

The samples were eluted in isocratic mode - 80% methanol (J. T. Baker, HPLC grade) and 20% ultrapure water - and the pH was adjusted to 3.0 with acetic acid. The flow rate of the mobile phase was 1.0 mL/min and the total time was 15 min, with 254 nm wavelength and 20 μL injection volume. The compartment of the chromatographic column oven was programmed to 30 °C. The equipment used was an HPLC (Thermo Scientific Dionex Ultimate 3000) equipped with a 0UV-VIS detector, an automatic injector with an oven and a four-channel pump with built-in degasser, and LUNA® 5 μm C18 (250 × 4.6 mm) column by Phenomenex [28].

The serum level of each sample was measured in triplicate, using the average of the concentrations found.

*Histopathological evaluation*

The histopathological examination of the intestines was performed in all animals. The intestines were collected and fixed in formalin for 48 h, then transferred to 70% alcohol and embedded in paraffin. Cuttings were performed, and afterwards, the slides were stained with hematoxylin and eosin (H&E).

## Results

### Minimum inhibitory concentrations (MICs)

**Figure 1.**
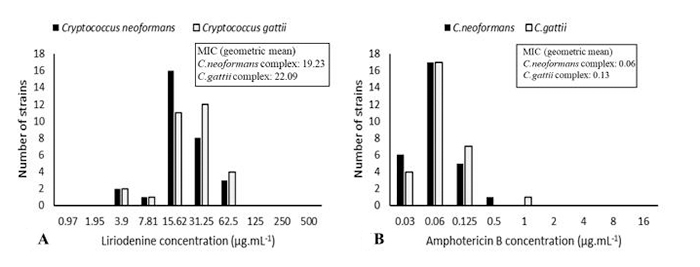
Minimum inhibitory concentrations (MICs) of liriodenine (LRD) and amphotericin B (AmB) for 30 strains of the *Cryptococcus neoformans* complex and 30 strains of the *Cryptococcus gattii* complex, determined by broth microdilution method. Results as median and interquartile intervals, as well as geometric mean. The Mann-Whitney U rank test. Comparison of MICs as to *Cryptococcus* complex, **(A)** LRD: *C. neoformans*: 15.62 [15.62; 31.25], *C. gattii*: 31.25 [15.62; 31.25], p = 0.30; **(B)** AmB: *C. neoformans*: 0.6 [0.06; 0.06], *C. gattii*: 0.06 [0.06; 0.13], p = 0.45.

The MIC of liriodenine ranged from 3.9 µg.mL^-1^ to 62.5 µg.mL^-1^ for *C. neoformans* as well as for *C. gattii* (Figure 1). MIC_90_ was 31.25 µg.mL^-1^ for both species. All the strains were inhibited to liriodenine and presented no differences in susceptibility regarding the species (*p* > 0.05). In addition, the low MICs of AmB did not differ between the species (*p* > 0.05). MIC_90_ was 0.125 µg.mL^-1^ for both species, and all strains showed high susceptibility to AmB. 

### Time-kill studies

**Figure 2.**
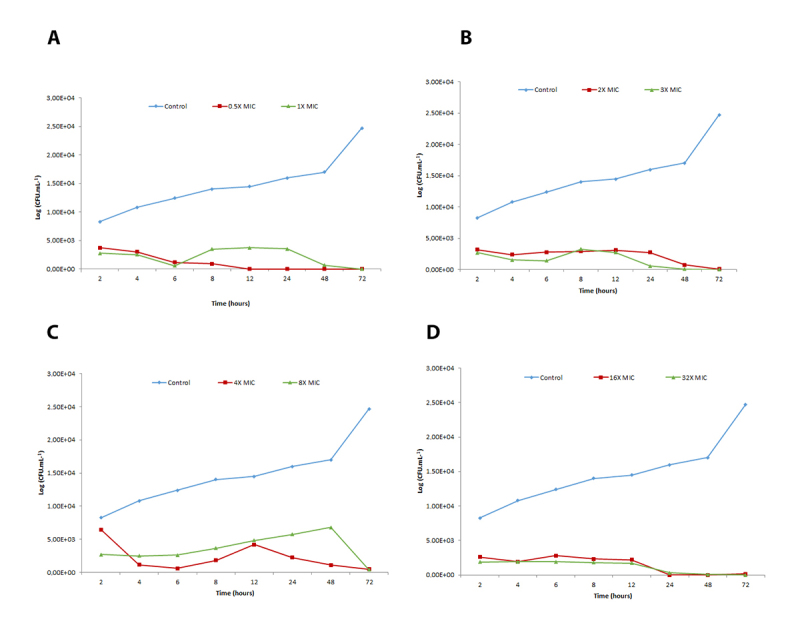
Time-kill curves for lireodenine against strain 17 of *Cryptococcus gattii* complex. Eight doubling dilutions are plotted, the highest concentration corresponds to 32 × MIC as measured with the EUCAST microdilution method, and the blue line represents growth in absence of lireodenine. The compound was added at timepoint 0 and monitored until 72 h. The limit of detection in the assay was 33 CFU/mL. CFU: colony forming units; MIC: minimum inhibitory concentration.

**Figure 3.**
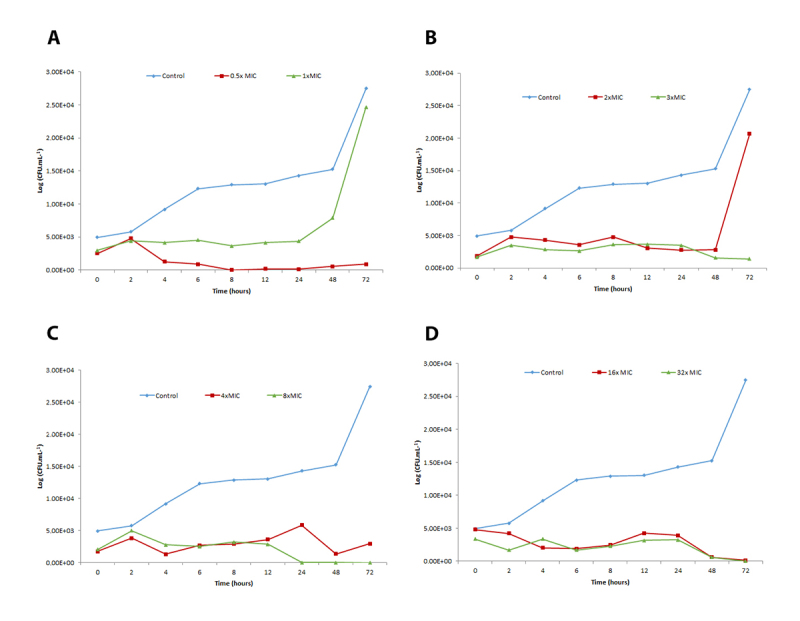
Time-kill curves for lireodenine against strain 57 of *Cryptococcus neoformans* complex. Eight doubling dilutions are plotted, the highest concentration corresponds to 32 × MIC as measured with the EUCAST microdilution method, and the blue line represents growth in absence of lireodenine. The compound was added at timepoint 0 and monitored until 72 h. The limit of detection in the assay was 33 CFU/mL. CFU: colony forming units; MIC: minimum inhibitory concentration.

**Figure 4.**
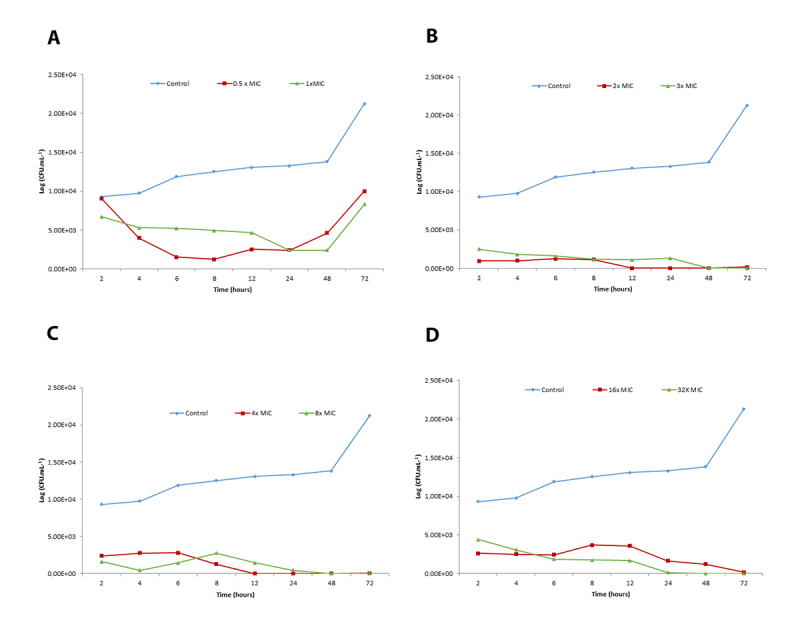
Time-kill curves for lireodenine against strain 32 of *Cryptococcus neoformans* complex. Eight doubling dilutions are plotted, the highest concentration corresponds to 32 × MIC as measured with the EUCAST microdilution method, and the blue line represents growth in absence of lireodenine. The compound was added at timepoint 0 and monitored until 72 h. The limit of detection in the assay was 33 CFU/mL. CFU: colony forming units; MIC: minimum inhibitory concentration.

Figures 2, 3 and 4 show time-kill curves for liriodenine using three strains exposed to four different agent concentrations and one untreated control of each strain. Liriodenine induced a fungicidal effect in all three strains, yet the onset of the fungicidal activity depended on the tested concentration and differed among the strains. All strains were killed to below the limit of detection (33 CFU.mL^−1^), at the highest liriodenine concentration (32-fold MIC). The strains experienced the most killing during the first 48 h at high antimicrobial concentrations. For strains 57 and 32, the fungicidal activity decreased at lower concentrations.

### Transmission electron microscopy

**Figure 5.**
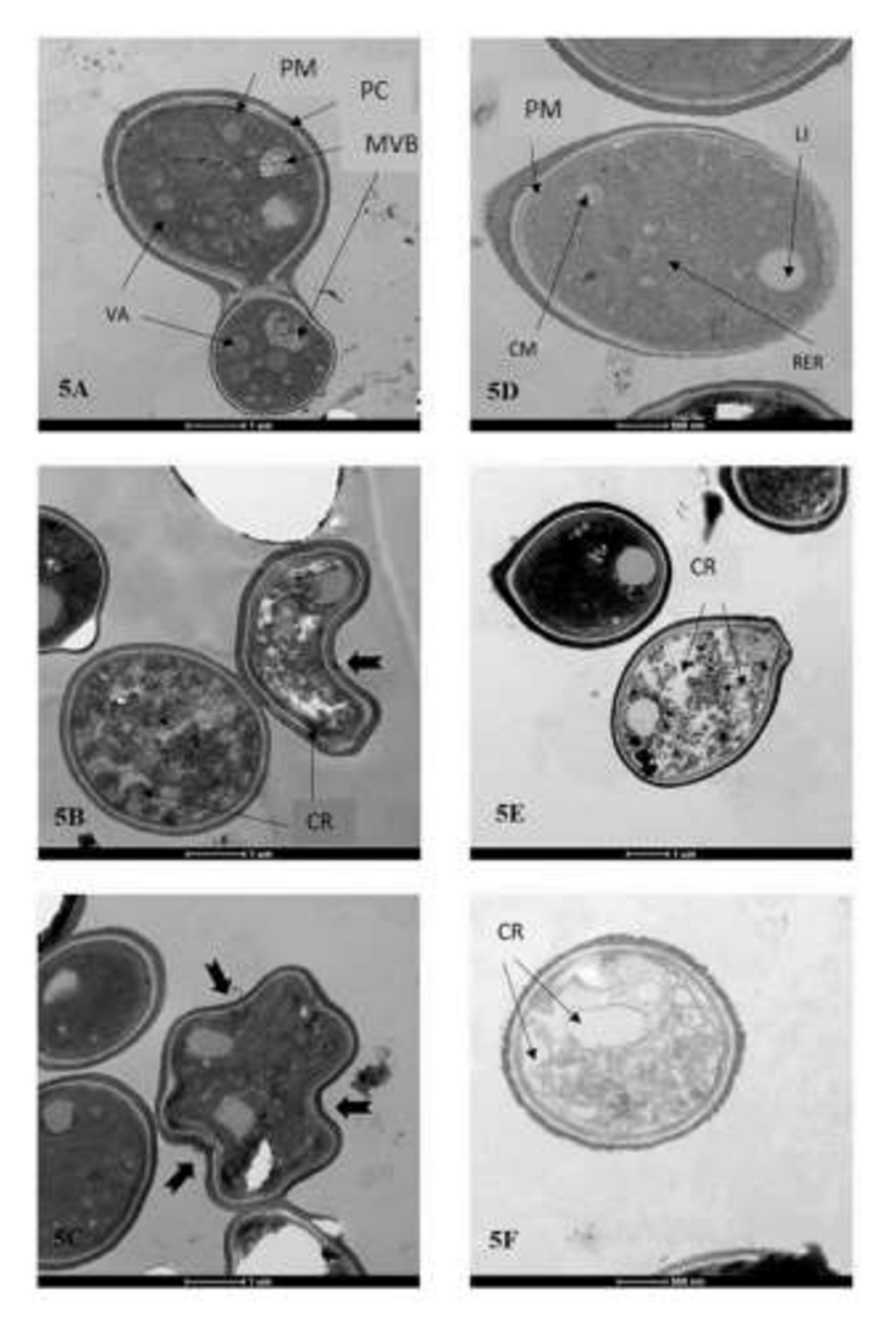
Transmission electron microscopy of *Cryptococcus neoformans* and *Cryptococcus gattii* species complexes. **(A)** Untreated *Cryptococcus neoformans* species complex cells. **(B, C)** Liriodenine-treated *Cryptococcus neoformans* species complex cells at a concentration of 31.25 µg.mL^-1^. **(D)** Untreated *Cryptococcus gattii* species complex cells. **(E, F)** Liriodenine-treated *Cryptococcus gattii* species complex cells at a concentration of 62.5 µg.mL^-1^. PM: plasma membrane; PC: polysaccharide capsule; MVB: multivesicular bodies; VA: vacuoles; LI: lipids; RER: rough endoplasmic reticulum; CR: cytoplasmic rarefaction; cell membrane irregularity.

Transmission electron microscopy (TEM) of untreated cryptococcal cells revealed round shape with regular contours, uniformly thick walls, and a polysaccharide capsule. The organelles were preserved and intact, enabling the detection of free ribosomes, a discrete rough endoplasmic reticulum, multivesicular bodies, vacuoles of a heterogeneous material and various sizes, and a few lipid droplets. Ligaments between cells and their buds were also demonstrated (Figures 5A and 5B).

However, liriodenine-treated cryptococcal cells presented significantly altered morphology. In both species complexes, the cytoplasm showed intense rarefaction and/or degradation, injury of organelles, vacuoles, or other structures, suggesting death of the microorganism (Figures 5B, 5C, 5E and 5F).

### *In vivo* evaluation of absorption and toxicity

*Clinical observation and histopathological analysis*

Prior to the administration of liriodenine, all animals had shiny hair, and had been calm eating regularly. Such conditions did not change with the administration of liriodenine.

Nevertheless, 12 h after the administration of liriodenine, the mice that had received the dose of 1.50 mg.kg^-1^ of body weight had abdominal distension; the laparotomy revealed the presence of large amounts of intestinal gas (Additional file 1). These findings were not observed in animals that received liriodenine at the dose of 0.75 mg.kg^-1^ of body weight.

**Figure 6.**
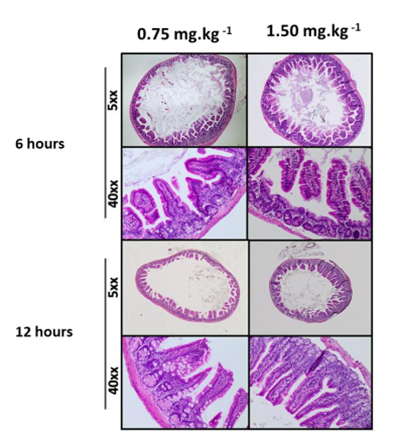
Intestinal histomorphology of BALB/c mice after administration of liriodenine by gavage. Note the absence of pathological changes at different doses (0.75 and 1.50 mg.kg^-1^) of liriodenine and different moments of euthanasia (6 and 12 h of administration) [H&E; 5XX].

The cross-sectional histological analyses of the intestines in all the animals, which were observed using light microscopy, showed no pathological alterations with either of the doses administered or in either of the two different moments of euthanasia (Figure 6).

*Serum dosage of liriodenine*

**Figure 7.**
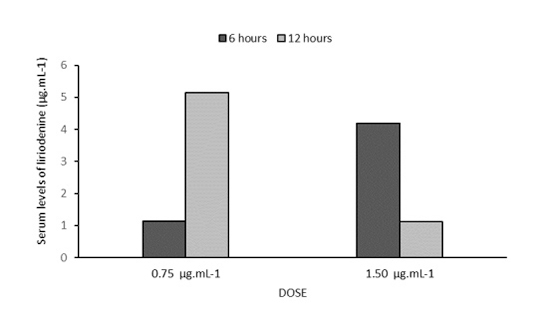
Serum levels of liriodenine were observed at 6 and 12 h after the administration by gavage of both doses of 0.75 and 1.50 mg.kg^-1^ of body weight. Data from one animal per dose at the time. The serum level is the average of three repetitions performed in each sample.

Liriodenine was detected in the serum of both mouse groups that had received it at the doses of 0.75 mg.kg^-1^ and 1.50 mg.kg^-1^ of body weight, confirming the absorption of this substance after administration by gavage (Figure 7).

However, serum levels of liriodenine showed different behavior due to the time of administration, according to the dose used in the mice: the ones that had received 0.75 mg.kg^-1^ presented an increase in concentration over time, while the opposite was observed in animals to which 1.50 mg.kg^-1^ had been administered.

## Discussion

In spite of the well-conducted studies on the treatment of cryptococcal meningitis, many questions still remain unanswered in the clinical practice, stimulating the investigation of new compounds, such as liriodenine [6-8, 29, 30]. The choice of this compound was based on some previous information: (a) liriodenine is extracted from *Annona mucosa*, a plant available in the Brazilian region where our university is located; (b) liriodenine extraction was performed in this university; (c) its chemical composition is known, making it easier to have a possible future production and modification of the molecule; (d) the literature review revealed some antimicrobial activity; (e) our previous study showed its activity on some fungi, mainly from the *Cryptococcus* genus [18]. 

Antifungal susceptibility testing (AST) has become a powerful tool in the choice and management of treatment of many systemic fungal diseases. Therefore, the protocols used in AST should be very well standardized and validated. The ones used in this study were RPMI 1640 buffered to a pH 7.0 with MOPS, incubation of time-kill samples at 35 °C with agitation, effect on antifungal carryover and evaluation for at least 24 hours compliant to Klepser et al. [23] standardization, being most of them confirmed by Zaragoza et al. [31]. In addition, the correlations between AST and clinical response are poor with MIC, increase a little with minimum fungicidal concentration (MFC), and are much better with the time-kill methodology [32], which was carried out in this study. As already described for the fungicidal drug AMB, isolates can show different profiles in the time-kill methodology that are impossible to identify only by MIC [24-26]. Future studies on pharmacokinetics and pharmacodynamics associated with time-kill data should improve the knowledge on the in vivo activity of liriodenine.

The present study demonstrated fungicidal activity of liriodenine on fungi of the *C. neoformans* and *C. gattii* species complexes, showing MIC values ranging from 3.9 to 62.5 µg.mL^-1^. Such results are similar to those obtained by Cruz et al. [33], who observed the activity of the synthetic 8-nitrohormone on eight strains of fungi from the *Cryptococcus* genus, with MICs of 40 µg.mL^-1^; however, the authors did not evaluate whether the compound presented a fungistatic or fungicidal effect.

The MICs of liriodenine observed in the present study were lower than those showed by the fatty acid methyl esters extracted from seeds of *Annona cornifolia* (> 500 µg.mL^-1^) [34], and they were also lower when compared to the essential oil and its major components linalool and geraniol extracted from *Ocimum basilicum* var. Maria Bonita, whose MICs ranged from 156 to 2500 µg.mL^-1^ [35]. Nevertheless, higher activity was found with maytenin and pristimerin, extracted from *Maytenus ilicifolia* (Celastraceae), when evaluated against five isolates of the *Cryptococcus* genus, showing MICs and MFCs ranging from 0.48 to 3.90 µg.mL^-1^ and from 0.97 to 7.81 µg.mL^-1^, respectively [36].

Punicalagin, a hydrosoluble tannin extracted from *Lafoensia pacari* A. St.-Hil (Lythraceae), presented a fungistatic effect on fungi of the *C. neoformans* complex, with MICs ranging from 0.5 to 4.0 µg.mL^-1^, which are values lower than those observed in the present study. However, its fungicidal activity was demonstrated at 256 µg.mL^-1^ [37].

The previous findings with liriodenine based on MFC [18] were confirmed by the present study, in which the time-kill methodology was carried out. In addition, the intense morphological alterations demonstrated by TEM - suggestive of killed cryptococcal cells - seem to justify liriodenine fungicidal activity. To the best of our knowledge, this is the second study showing the effect of an antifungal compound on cryptococcal cells using TEM. 

The mechanism of action of liriodenine has yet to be demonstrated. The results of this study could be taken into consideration, contributing to fill this gap. The *in vitro* and *in vivo* inhibition of the topoisomerase II enzyme of the DNA, alike the quinolones, suggest an interference in the RNA and protein synthesis [38]. Furthermore, melanin, produced by the action of phenol oxidase on L-DOPA [39, 40], is a virulence factor for fungi of the *Cryptococcus* genus for protecting them from oxidative stress, phagocytosis, the action of antifungal compounds, as well as for modifying the host immune response [41-46]. As liriodenine inhibits the melanin synthesis in PC-12 cells, this anti-melanin activity could play a role in its anticryptococcal activity [47]. Finally, liriodenine causes an imbalance in the iron cell homeostasis, leading to the accumulation of the mitochondrial iron, a decrease in the number of iron enzymes, and an increase in the oxidative stress, which causes fungal death [48].

Ultrastructural studies of antifungal compound actions on *Cryptococcus* cells are scarce [49-51]. Subinhibitory concentrations, from 0.125 to 0.5 of MIC either of AmB or of fluconazole, alter cell and capsule size, and cell shape at scanning electron microscopy [49]. Terbinafine, a fungistatic compound, causes detachment of the cell membrane from the cell wall, which probably results in membrane impairment, as well as increased cytoplasmic vacuoles that could account for the lipid accumulation and mitochondrial swelling [51]. Some of these findings, including irregularity of cytoplasmic membranes and cytoplasmic vacuoles, are also observed with liriodenine, a fungicidal compound.

The evaluation of liriodenine serum levels after gavage administration suggests better absorption with the lower dose used, 0.75 mg.kg^-1^, which may be justified by the gas formation determined by the dose of 1.50 mg.kg^-1^. Thus, the abdominal distension due to gas formation could lead to a reduction in the absorption of liriodenine since the histopathological evaluation with the H&E staining of the intestinal fragments did not reveal any organic lesion. This result prompts the development of further studies on liriodenine or its derivatives as an anticryptococcal compound.

The potential weakness of this study is MIC_90_ higher than that observed with AmB. Such finding suggests the investigation of liriodenine derivatives with the same fungicidal activity but with lower MICs, which is ongoing. The best derivatives will progress into the determination of the mechanism of action, pharmacokinetic studies, and combination with other antifungal compounds. 

## Conclusions

The present *in vitro* studies of liriodenine on fungi of *C. neoformans* and *C. gattii* species complexes demonstrated intense structural alterations of the yeast cells and fungicidal activity when assessed by time-kill methodology. These results suggest that this molecule can be a promising prototype of anticryptococcal compounds. Investigations on modifications of the liriodenine molecule to improve its pharmacological properties and experimental cryptococcal infection are currently ongoing for evaluating the safety and efficacy of this antifungal compound and its derivatives. 

## Supplementary material

The following online material is available for this article:

Additional file 1. BALB/c mice receiving liriodenine by gavage at a concentration of 1.50 mg.kg^-1^. The euthanasia was performed 12 h after administration of liriodenine. **(A1-B1)** Mice during the euthanasia. **(A2-B2)** Intestines removed from the dead animals.
