# Supplementary material for: Antifungal activity of liriodenine on clinical strains of Cryptococcus neoformans and Cryptococcus gattii species complexes
Source: J Venom Anim Toxins Incl Trop Dis. 2022 Sep 5;28:e20220006. doi: 10.1590/1678-9199-JVATITD-2022-0006 (PMC9469771; doi:10.1590/1678-9199-JVATITD-2022-0006)
Supplement: Additional file 1. [file 1678-9199-jvatitd-28-e20220006-s1.pdf]

J Venom Anim Toxins incl Trop Dis
https://doi.org/10.1590/1678-9199-JVATITD-2022-0006

Supplementary Material to "Antifungal activity of liriodenine on clinical strains of *Cryptococcus neoformans* and *Cryptococcus gattii* species complexes"

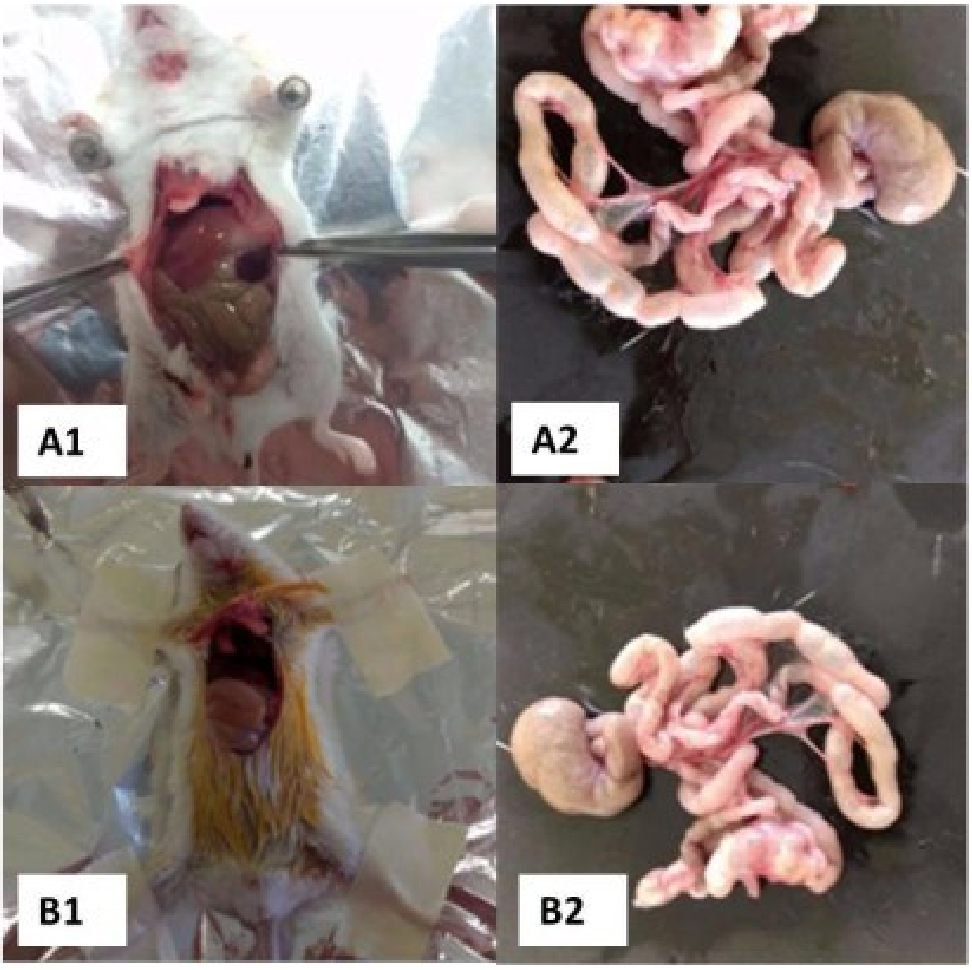

Additional file 1. BALB/c mice receiving liriodenine by gavage at a concentration of 1.50 mg.kg^{-1}. The euthanasia was performed 12 h after administration of liriodenine. **(A1-B1)** Mice during the euthanasia. **(A2-B2)** Intestines removed from the dead animals.
